# Supplementary material for: Closing the gap to effective gene drive in Aedes aegypti by exploiting germline regulatory elements
Source: Nat Commun. 2023 Jan 20;14:338. doi: 10.1038/s41467-023-36029-7 (PMC9860013; doi:10.1038/s41467-023-36029-7)
Supplement: Supplementary file 1 — Supplementary Information [file 41467_2023_36029_MOESM1_ESM.pdf]

Supplementary information

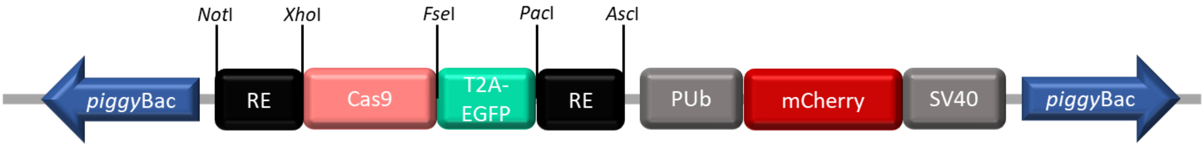

line	Upstream RE	Downstream RE
Sds3-Cas9 (AGG1208)	AaegL5_1:58240291-58242249	AegL5_1:58260607-58260914, additional P10 3'UTR
zpg2k-Cas9 (AGG1448)	AaegL5_2:84861078-84863147	AaegL5_2:84865496-84865750, additional P10 3'UTR

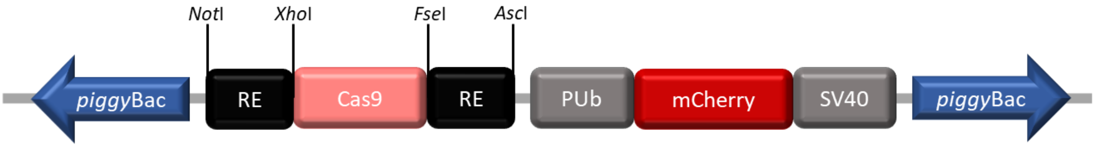

line	Upstream RE	Downstream RE
nos-Cas9 (AGG1547)	AaegL5_1:228706-229655	AegL5_1:240375-241057
shu-Cas9 (AGG1551)	AaegL5_3:281559539-281557722	AaegL5_3:281556144-281556028
Ewal-Cas9 (AGG1552)	AaegL5_145302724-145300725	AaegL5_3:145288952-145287807
zpg4kb-Cas9 (AGG1687)	AaegL5_2:84858940-84863147	AaegL5_2:84865496-84865750

Supplementary Figure S1. Diagram of Cas9 constructs. Full depiction of the piggyBac construct used for transgenesis. Restriction enzymes used for cloning are indicated. Tables list the regions of the genome amplified for use as the promoter (Upstream RE) and 3'UTR (downstream RE).

Supplementary Table S1. Microinjection of embryos to generate Cas9-expressing lines.

Construct	No. of injected embryos	G_0 survivors (%)	G_1 larvae screened	Positive G_1 larvae	No. of positive pools (pool ID)	isolines analysed
Ewald-Cas9 (AGG1552)	323	24 (7.4)	1916	34	1/3 (C)	C1, C3
nos-Cas9 (AGG1547)	346	73 (21.1)	2710	122	6/8 (A, B, C, D, H, J)	A2, C2, D1
sds3-Cas9 (AGG1208)	990	161 (16.3)	4664	464	8/8 (A, B, C, D, E, F, G, H)	A1, E1, G1
shu-Cas9 (AGG1551)	324	92 (28.3)	3444	175	5/10 (A, B, C, I, K)	A1, B1, C1, C2
*zpg*2kb-Cas9 (AGG1448)	442	55 (12.4)	13745	46	3/6 (C, D, E)	C1
*zpg*4kb-Cas9 (AGG1687)	371	27 (7.3)	1189	59	1/3 (C)	C1, C2

Supplementary Table S2. Heterozygous viability of *sds3*G1-Cas9

(♀) WT X *sds3*G1-Cas9/+ (♂)	Live	Dead	% Dead by sex	% Dead by type	Difference in %
♂ *sds3*G1-Cas9	276	63	18.58	16.39	0.23
♀ *sds3*G1-Cas9	270	44	14.01		
♂ WT	280	52	15.66	16.61	
♀ WT	242	52	17.69		

Supplementary Table S3. Homozygous viability of *sds3*-Cas9

*sds3*G1-Cas9/+ X *sds3*G1-Cas9/+					
	-	%	+	%	Total
Larvae	187	27.9	484	72.1	671
Adults	166	27.3	443	72.7	609
Male adults	87	52.4	213	48.1	300
Females adults	79	47.6	230	51.9	309

Supplementary Table S4. Inheritance rate of *kmo*^{sgRNAs}

F_0	Inheritance rate of *kmo*^{sgRNAs} (%)<br>No. of larvae screened	
	F_1 transhet female	F_1 transhet male
♀ *kmo*^{sgRNAs} X ♂ *sds3*G1-Cas9	89.8<br>421	69.6<br>1265
♀ *kmo*^{sgRNAs};*sds3*G1-Cas9 X ♂ LVP	92.0<br>162	85.3<br>868
♀ LVP X ♂ *kmo*^{sgRNAs};*sds3*G1-Cas9	97.6<br>455	97.0<br>1804
Total inheriting	971	3371
Total larvae screened	1038	3937
Overall inheritance (%)	93.5	85.6
Homing rate (%)	87.1	71.2

Supplementary Table S5. Genomic locus of Cas9 transgene integrations.

Isoline	Genomic sequence ID (VectorBase)	Nucleotide position	region
*shu*A-Cas9	AaegL5_3	380,318,728-380,318,729	intronic between exon 5 and 6 of AAEL000961 (unspecified product) and exon 1 and 2 of AAEL000943 (unspecified product)
*shu*B-Cas9	AaegL5_1	194,389,299-194,389,300	intergenic between AAEL027430 (unspecified product) and AAEL010001 (unspecified product)
*shu*C1-Cas9	AaegL5_3	191,252,140-191,252,141	intronic between exon 1 and 2 of AAEL007171 (Protein phosphatase 2c)
*shu*C2-Cas9	AaegL5_3	18,109,307-18,109,308	within exon 1 of AAEL020025 unspecified lncRNA
*sds3*A-Cas9*	AaegL5_3	207,249,192-207,249,193	intergenic between AAEL023012 (unspecified lncRNA) and AAEL007794 (defective proboscis extension response)
*sds3*E-Cas9*	AaegL5_3	312,663,232-312,663,233	intronic between exon 1 and 2 of the 5'UTR of AAEL023098 (unspecified product)
*sds*3G-Cas9	AaegL5_3	338,343,812-338,343,813	intronic between the first and second coding exons of AAEL002469 (endophilin a)

*these insertion sites have not been confirmed by PCR and Sanger sequencing

Supplementary Table S6. List of oligonucleotides.

Oligo ID	Function	Sequence (5'-3')
LA179<br>LA191	Adapter for MspI-digested DNA	GTGTAGCGTGAAGACGACAGAAAGGGCGTGGTGCGGAGGGCGGTG<br>CGCACCGCCCTCCG
LA179<br>LA192	Adapter for BamHI-digested DNA	GTGTAGCGTGAAGACGACAGAAAGGGCGTGGTGCGGAGGGCGGTG<br>GATCCACCGCCCTCCG
LA179<br>LA1703	Adapter for NcoI-digested DNA	GTGTAGCGTGAAGACGACAGAAAGGGCGTGGTGCGGAGGGCGGTG<br>CATGCACCGCCCTCCG
LA187<br>LA183	5' end primary adapter ligation-mediated PCR	GTGTAGCGTGAAGACGACAGAA<br>CAGTGACACTTACCGCATTGACAAG
LA187<br>LA182	5' end secondary adapter ligation-mediated PCR	GTGTAGCGTGAAGACGACAGAA<br>GGCGACTGAGATGTCCTAAATGCAC
LA187<br>LA184	3' end primary adapter ligation-mediated PCR	GTGTAGCGTGAAGACGACAGAA<br>CAGACCGATAAAACACATGCGTCA
LA187<br>LA1064	3' end secondary adapter ligation-mediated PCR	GTGTAGCGTGAAGACGACAGAA<br>ACGCATGATTATCTTTAACGTACG
LA1084<br>LA1350 (nest)	sds3 5'RACE	TGTGCTGTTCGTATGGTTCCGGATGG<br>TCGTCCAGCAAAAGAACCAACTGCCCAG
LA1082<br>LA1083 (nest)	sds3 3'RACE	ACGTCGACCTAATGAACCGCTTCCG<br>GGGCAGTTGGTTCTTTTGCTGGACG
LA2956<br>LA2955 (nest)	ewald 5'RACE	CGCTTGAGGCCACGTCCCGTCGATGAGAATGAG<br>GGGAACGAGTGGCTGCGGTGGCAGCGCCGAAC
LA2958<br>LA2957 (nest)	ewald 3'RACE	CTCATTCTCATCGACGGGACGTGGCCTCAAGCG<br>GCCATCGAAGCGCTGAGCATTCTGGAGCGGGAC
LA2952<br>LA2951 (nest)	shu 5'RACE	CGGTCCCAGGGATTCATGATCTTACGGTGGGC<br>GGTGCTGATCTGGAATTGGGCTCCATGTTCCCCG
LA2954<br>LA2953 (nest)	shu 3'RACE	CGATGAAGAAGAACCTGGAGGAGTTTGTGGCGG<br>GGGCCTCACCACCAAGGAGATGGACATCGTCCAGG
LA2963<br>LA2964 (nest)	zpg 5'RACE	GGAAGGCGAAGATTTTCTCGTTGACGATGTTCTGCGGCAGA<br>CCCAGTGTCCAACAGAAGCTGTGTAGACTGGCCCT
LA2965<br>LA2966 (nest)	zpg 3'RACE	CGCCTTCCTGTGGCTTTGGTTCATTGTGCTGGCCG<br>CCTGCCGCAGCGTTCGGTTCCAACTGTTGTTCAGC
LA2811<br>LA2812	ewald promoter	TTTTGCGGCCGCACTGTTCAACTGATTCTTCAAGAATACC<br>TTTTCTCGAGTTTCACTATTCAAAAGGACTTTCTTTCAAATCTCACC
LA4066<br>LA4077	ewald 3'UTR	TTTTGGCCGGCCAGGCAAAAAAGAAAAAGTCTCCGGGGGGTTGGTGTGTGTTTCTGTCAG<br>TTTTGGCGCGCCCTTCAGACCAAAATCAGTTGAGAATTAAG
LA3769<br>LA3770	nos promoter	TTTTGCGGCCGCATCACTATCAAACCCCTAAAGACA<br>TTTTCTCGAGTTCCGCTTCACGTTTGCACCGCTTTATGACC

LA4072<br>LA4083	nos 3’UTR	TTTTGGCCGGCCAGGCAAAAAAGAAAAAGTTGTTTTTTTTTATGAAATGTTTCTGACTGTGAC<br>TTTTGGCGCGCCACCAGACATCCGTTTAAGCTGAACCAC
LA1798<br>LA1728	sds3 promoter	TTTTGCGGCCGCTCTGTTTGAATATGTTTCCGAGAA<br>TTTTCTCGAGTTTCCGCGACAAAAACACAGA
LA1739<br>LA1740	sds3 3’UTR	TTTTTTAATTAAGGAAACAAGGATCTCAACTCTCGAGC<br>TTTTGCGATCGCCCTCGAGCTTCTTAGGTACAATTGTAAAACATAGTT
LA2807<br>LA2808	shu promoter	TTTTGCGGCCGCTCTTCACAACTCCAAATTCTACCACATCTTCGG<br>TTTTCTCGAGTGTTTGAAGATTTAAGTTTTTGGAATGAAAAAGATGG
LA4063<br>LA4076	shu 3’UTR	TTTTGGCCGGCCAGGCAAAAAAGAAAAAGGGAAACAAGGATCTCAACTCTCGAGC<br>TTTTGGCGCGCCTATATGATTGAGTAGGTAGTCATTTATTTCAG
LA2803<br>LA2804	zpg2kb<br>promoter	TTTTGCGGCCGCCAGCTGATTTGAGACGTCATTTGAATAGGCC<br>TTTTCTCGAGGATGATTTAGGGGTTTGAACGTTTCTTTTTTCGGTA
LA3945<br>LA3946	zpg4kb<br>promoter	ACGGTCACGGCGGGCATGTCGACGCAGCGTAATGATACTGCGCTATTTATG<br>TCCGTCGTGGTCCTTATAGTCCATCGATGATTTAGGGGTTTGAACG
LA2805<br>LA2806	zpg 3’UTR<br>(2kb pro)	TTTTTTAATTAATCGATAAAAGTATCGTCCTAAGACTTATTCAG<br>TTTTTTTTGCGATCGCTTATGTTTCACATACTTTATTCTATATTTGC
LA4067<br>LA4078	zpg 3’UTR<br>(4kb pro)	TTTTGGCCGGCCAGGCAAAAAAGAAAAAGTCGATAAAAGTATCGTCCTAAGACTTATTCAG<br>TTTTGGCGCGCCCTTATGTTTCACATACTTTATTCTATATTTGC

Oligos used to make adapters complement at regions highlighted in yellow.

Supplementary Note 1. Structure and associated parameter values used for the mathematical model.

Mathematical model

Here we use a deterministic, discrete generation model of population genetics within a large and panmictic population – similar to that used in our previous work[1]. The assumptions upon which this model is based are well founded and have previously been discussed at length[2–8] so for brevity that discussion need not be repeated here. This model uses recursion equations to calculate the genotype frequencies in a generation ($i+1$) based on those in the previous generation (i), giving equations of the form

$$\begin{aligned} M_1^{i+1} = \frac{\Omega_{M1}}{2}\Big(& M_1^iF_1^i + 0.5\,M_1^iF_2^i + 0.5\,M_1^iF_4^i + 0.25\,M_1^iF_5^i(1-\Phi_F) + 0.5\,M_2^iF_1^i + 0.25\,M_2^iF_2^i \\ &+ 0.25\,M_2^iF_4^i + 0.125\,M_2^iF_5^i\,(1-\Phi_F) + 0.5\,M_4^iF_1^i + 0.25\,M_4^iF_2^i + 0.25\,M_4^iF_4^i \\ &+ 0.125\,M_4^iF_5^i\,(1-\Phi_F) + 0.25\,M_5^i(1-\Phi_M)F_1^i + 0.125\,M_5^i(1-\Phi_M)F_2^i \\ &+ 0.125\,M_5^i(1-\Phi_M)F_4^i + 0.0625\,M_5^i(1-\Phi_M)F_5^i(1-\Phi_F)\Big)\,/\,\hat{\Omega}\,, \end{aligned}$$

$$\begin{aligned} M_2^{i+1} = \frac{\Omega_{M2}}{2}\big(& 0.5\,M_1^iF_2^i + M_1^iF_3^i + 0.25\,M_1^iF_5^i(1-\Phi_F) + 0.5\,M_1^iF_6^i + 0.5\,M_2^iF_1^i + 0.5\,M_2^iF_2^i \\ &+ 0.5\,M_2^iF_3^i + 0.25\,M_2^iF_4^i + 0.25\,M_2^iF_5^i(1-\Phi_F) + 0.25\,M_2^iF_6^i + M_3^iF_1^i + 0.5\,M_3^iF_2^i \\ &+ 0.5\,M_3^iF_4^i + 0.25\,M_3^iF_5^i(1-\Phi_F) + 0.25\,M_4^iF_2^i + 0.5\,M_4^iF_3^i + 0.125\,M_4^iF_5^i(1-\Phi_F) \\ &+ 0.25\,M_4^iF_6^i + 0.25\,M_5^i(1-\Phi_M)F_1^i + 0.25\,M_5^i(1-\Phi_M)F_2^i + 0.25\,M_5^i(1-\Phi_M)F_3^i \\ &+ 0.125\,M_5^i(1-\Phi_M)F_4^i + 0.125\,M_5^i(1-\Phi_M)F_5^i(1-\Phi_F) + 0.125\,M_5^i(1-\Phi_M)F_6^i \\ &+ 0.5\,M_6^iF_1^i + 0.25\,M_6^iF_2^i + 0.25\,M_6^iF_4^i + 0.125\,M_6^iF_5^i(1-\Phi_F) + 0.5\,M_1^iF_5^i\Phi_F \\ &+ 0.25\,M_2^iF_5^i\Phi_F + 0.25\,M_4^iF_5^i\Phi_F + 0.125\,M_5^iF_5^i\Phi_F(1-\Phi_M) + 0.5\,M_5^iF_1^i\Phi_M \\ &+ 0.25\,M_5^iF_2^i\Phi_M + 0.25\,M_5^iF_4^i\Phi_M + 0.125\,M_5^iF_5^i\Phi_M(1-\Phi_F) \\ &+ 0.125\,(1-\Phi_M)M_5^i\Phi_FF_8^i\big)/\,\hat{\Omega}\,, \end{aligned}$$

$$\begin{aligned} M_3^{i+1} = \frac{\Omega_{M3}}{2}\big(& 0.25\,M_2^iF_2^i + 0.5\,M_2^iF_3^i + 0.125\,M_2^iF_5^i(1-\Phi_F) + 0.25\,M_2^iF_6^i + 0.5\,M_3^iF_2^i + M_3^iF_3^i \\ &+ 0.25\,M_3^iF_5^i(1-\Phi_F) + 0.5\,M_3^iF_6^i + 0.125\,M_5^i(1-\Phi_M)F_2^i + 0.25\,M_5^i(1-\Phi_M)F_3^i \\ &+ 0.0625\,M_5^i(1-\Phi_M)F_5^i(1-\Phi_F) + 0.125\,M_5^i(1-\Phi_M)F_6^i + 0.25\,M_6^iF_2^i \\ &+ 0.5\,M_6^iF_3^i + 0.125\,M_6^iF_5^i(1-\Phi_F) + 0.25\,M_6^iF_6^i + 0.25\,M_2^iF_5^i\Phi_F + 0.5\,M_3^iF_5^i\Phi_F \\ &+ 0.125\,M_5^iF_5^i\Phi_F(1-\Phi_M) + 0.25\,M_6^iF_5^i\Phi_F + 0.25\,M_5^iF_2^i\Phi_M + 0.5\,M_5^iF_3^i\Phi_M \\ &+ 0.125\,M_5^iF_5^i\Phi_M(1-\Phi_F) + 0.25\,M_5^iF_6^i\Phi_M + 0.25\,M_5^iF_5^i\Phi_M\Phi_F \\ &+ 0.125\,(1-\Phi_M)M_5^i\Phi_FF_8^i\big)/\,\hat{\Omega}\,, \end{aligned}$$

$$\begin{aligned} M_4^{i+1} = \frac{\Omega_{M4}}{2}\Big(& 0.5\,M_1^iF_4^i + 0.25\,M_1^iF_5^i(1-\Phi_F) + M_1^iF_7^i + 0.5\,M_1^iF_8^i(1-\Phi_F) + 0.25\,M_2^iF_4^i \\ &+ 0.125\,M_2^iF_5^i(1-\Phi_F) + 0.5\,M_2^iF_7^i + 0.25\,M_2^iF_8^i(1-\Phi_F) + 0.5\,M_4^iF_1^i + 0.25\,M_4^iF_2^i \\ &+ 0.5\,M_4^iF_4^i + 0.25\,M_4^iF_5^i(1-\Phi_F) + 0.5\,M_4^iF_7^i + 0.25\,M_4^iF_8^i(1-\Phi_F) \\ &+ 0.25\,M_5^i(1-\Phi_M)F_1^i + 0.125\,M_5^i(1-\Phi_M)F_2^i + 0.25\,M_5^i(1-\Phi_M)F_4^i \\ &+ 0.125\,M_5^i(1-\Phi_M)F_5^i(1-\Phi_F) + 0.25\,M_5^i(1-\Phi_M)F_7^i \\ &+ 0.125\,M_5^i(1-\Phi_M)F_8^i(1-\Phi_F) + M_7^iF_1^i + 0.5\,M_7^iF_2^i + 0.5\,M_7^iF_4^i \\ &+ 0.25\,M_7^iF_5^i(1-\Phi_F) + 0.5\,M_8^i(1-\Phi_M)F_1^i + 0.25\,M_8^i(1-\Phi_M)F_2^i \\ &+ 0.25\,M_8^i(1-\Phi_M)F_4^i + 0.125\,M_8^i(1-\Phi_M)F_5^i(1-\Phi_F)\Big)\,/\,\hat{\Omega}\,, \end{aligned}$$

$$
\begin{aligned}
M_5^{i+1} = \frac{\Omega_{M5}}{2}\Big(&0.25\, M_1^i F_5^i (1-\Phi_F) + 0.5\, M_1^i F_6^i + 0.5\, M_1^i F_8^i (1-\Phi_F) + M_1^i F_9^i + 0.25\, M_2^i F_4^i \\
&+ 0.25\, M_2^i F_5^i (1-\Phi_F) + 0.25\, M_2^i F_6^i + 0.5\, M_2^i F_7^i + 0.5\, M_2^i F_8^i (1-\Phi_F) + 0.5\, M_2^i F_9^i \\
&+ 0.5\, M_3^i F_4^i + 0.25\, M_3^i F_5^i (1-\Phi_F) + M_3^i F_7^i + 0.5\, M_3^i F_8^i (1-\Phi_F) + 0.25\, M_4^i F_2^i \\
&+ 0.5\, M_4^i F_3^i + 0.25\, M_4^i F_5^i (1-\Phi_F) + 0.5\, M_4^i F_6^i + 0.25\, M_4^i F_8^i (1-\Phi_F) + 0.5\, M_4^i F_9^i \\
&+ 0.25\, M_5^i (1-\Phi_M) F_1^i + 0.25\, M_5^i (1-\Phi_M) F_2^i + 0.25\, M_5^i (1-\Phi_M) F_3^i \\
&+ 0.25\, M_5^i (1-\Phi_M) F_4^i + 0.25\, M_5^i (1-\Phi_M) F_5^i (1-\Phi_F) + 0.25\, M_5^i (1-\Phi_M) F_6^i \\
&+ 0.25\, M_5^i (1-\Phi_M) F_7^i + 0.25\, M_5^i (1-\Phi_M) F_8^i (1-\Phi_F) + 0.25\, M_5^i (1-\Phi_M) F_9^i \\
&+ 0.5\, M_6^i F_1^i + 0.25\, M_6^i F_2^i + 0.5\, M_6^i F_4^i + 0.25\, M_6^i F_5^i (1-\Phi_F) + 0.5\, M_6^i F_7^i \\
&+ 0.25\, M_6^i F_8^i (1-\Phi_F) + 0.5\, M_7^i F_2^i + M_7^i F_3^i + 0.25\, M_7^i F_5^i (1-\Phi_F) + 0.5\, M_7^i F_6^i \\
&+ 0.5\, M_8^i (1-\Phi_M) F_1^i + 0.5\, M_8^i (1-\Phi_M) F_2^i + 0.5\, M_8^i (1-\Phi_M) F_3^i \\
&+ 0.25\, M_8^i (1-\Phi_M) F_4^i + 0.25\, M_8^i (1-\Phi_M) F_5^i (1-\Phi_F) 0.25\, M_8^i (1-\Phi_M) F_6^i + M_9^i F_1^i \\
&+ 0.5\, M_9^i F_2^i + 0.5\, M_9^i F_4^i + 0.25\, M_9^i F_5^i (1-\Phi_F) + 0.5\, M_1^i F_5^i \Phi_F + M_1^i F_8^i \Phi_F \\
&+ 0.25\, M_2^i F_5^i \Phi_F + 0.5\, M_2^i F_8^i \Phi_F + 0.5\, M_4^i F_5^i \Phi_F + 0.5\, M_4^i F_8^i \Phi_F \\
&+ 0.25\, M_5^i F_5^i \Phi_F (1-\Phi_M) + 0.25\, M_5^i F_8^i \Phi_F (1-\Phi_M) + 0.5\, M_7^i F_5^i \Phi_F \\
&+ 0.25\, M_8^i F_5^i \Phi_F (1-\Phi_M) + 0.5\, M_5^i F_1^i \Phi_M + 0.25\, M_5^i F_2^i \Phi_M + 0.5\, M_5^i F_4^i \Phi_M \\
&+ 0.25\, M_5^i F_5^i \Phi_M (1-\Phi_F) + 0.5\, M_5^i F_7^i \Phi_M + 0.25\, M_5^i F_8^i \Phi_M (1-\Phi_F) + M_8^i F_1^i \Phi_M \\
&+ 0.5\, M_8^i F_2^i \Phi_M + 0.5\, M_8^i F_4^i \Phi_M + 0.25\, M_8^i F_5^i \Phi_M (1-\Phi_F)\Big) / \hat{\Omega}\,,
\end{aligned}
$$

$$
\begin{aligned}
M_6^{i+1} = \frac{\Omega_{M6}}{2}\big(&0.125\, M_2^i F_5^i (1-\Phi_F) + 0.25\, M_2^i F_6^i + 0.25\, M_2^i F_8^i (1-\Phi_F) + 0.5\, M_2^i F_9^i \\
&+ 0.25\, M_3^i F_5^i (1-\Phi_F) + 0.5\, M_3^i F_6^i + 0.5\, M_3^i F_8^i (1-\Phi_F) + M_3^i F_9^i \\
&+ 0.125\, M_5^i (1-\Phi_M) F_2^i + 0.25\, M_5^i (1-\Phi_M) F_3^i + 0.125\, M_5^i (1-\Phi_M) F_5^i (1-\Phi_F) \\
&+ 0.25\, M_5^i (1-\Phi_M) F_6^i + 0.125\, M_5^i (1-\Phi_M) F_8^i (1-\Phi_F) + 0.25\, M_5^i (1-\Phi_M) F_9^i \\
&+ 0.25\, M_6^i F_2^i + 0.5\, M_6^i F_3^i + 0.25\, M_6^i F_5^i (1-\Phi_F) + 0.5\, M_6^i F_6^i + 0.25\, M_6^i F_8^i (1-\Phi_F) \\
&+ 0.5\, M_6^i F_9^i + 0.25\, M_8^i (1-\Phi_M) F_2^i + 0.5\, M_8^i (1-\Phi_M) F_3^i \\
&+ 0.125\, M_8^i (1-\Phi_M) F_5^i (1-\Phi_F) + 0.25\, M_8^i (1-\Phi_M) F_6^i + 0.5\, M_9^i F_2^i + M_9^i F_3^i \\
&+ 0.25\, M_9^i F_5^i (1-\Phi_F) + 0.5\, M_9^i F_6^i + 0.25\, M_2^i F_5^i \Phi_F + 0.5\, M_2^i F_8^i \Phi_F + 0.5\, M_3^i F_5^i \Phi_F \\
&+ M_3^i F_8^i \Phi_F + 0.25\, M_5^i F_5^i \Phi_F (1-\Phi_M) + 0.25\, M_5^i F_8^i \Phi_F (1-\Phi_M) + 0.5\, M_6^i F_5^i \Phi_F \\
&+ 0.5\, M_6^i F_8^i \Phi_F + 0.25\, M_8^i F_5^i \Phi_F (1-\Phi_M) + 0.5\, M_9^i F_5^i \Phi_F + 0.25\, M_5^i F_2^i \Phi_M \\
&+ 0.5\, M_5^i F_3^i \Phi_M + 0.25\, M_5^i F_5^i \Phi_M (1-\Phi_F) + 0.5\, M_5^i F_6^i \Phi_M + 0.25\, M_5^i F_8^i \Phi_M (1-\Phi_F) \\
&+ 0.5\, M_5^i F_9^i \Phi_M + 0.5\, M_8^i F_2^i \Phi_M + M_8^i F_3^i \Phi_M + 0.25\, M_8^i F_5^i \Phi_M (1-\Phi_F) + 0.5\, M_8^i F_6^i \Phi_M \\
&+ 0.5\, M_5^i F_5^i \Phi_M \Phi_F + 0.5\, M_5^i F_8^i \Phi_M \Phi_F + 0.5\, M_8^i F_5^i \Phi_M \Phi_F\big) / \hat{\Omega}\,,
\end{aligned}
$$

$$
\begin{aligned}
M_7^{i+1} = \frac{\Omega_{M7}}{2}\Big(&0.25\, M_4^i F_4^i + 0.125\, M_4^i F_5^i (1-\Phi_F) + 0.5\, M_4^i F_7^i + 0.25\, M_4^i F_8^i (1-\Phi_F) \\
&+ 0.125\, M_5^i (1-\Phi_M) F_4^i + 0.0625\, M_5^i (1-\Phi_M) F_5^i (1-\Phi_F) + 0.25\, M_5^i (1-\Phi_M) F_7^i \\
&+ 0.125\, M_5^i (1-\Phi_M) F_8^i (1-\Phi_F) + 0.5\, M_7^i F_4^i + 0.25\, M_7^i F_5^i (1-\Phi_F) + M_7^i F_7^i \\
&+ 0.5\, M_7^i F_8^i (1-\Phi_F) + 0.25\, M_8^i (1-\Phi_M) F_4^i + 0.125\, M_8^i (1-\Phi_M) F_5^i (1-\Phi_F) \\
&+ 0.5\, M_8^i (1-\Phi_M) F_7^i + 0.25\, M_8^i (1-\Phi_M) F_8^i (1-\Phi_F)\Big) / \hat{\Omega}\,,
\end{aligned}
$$

$$
\begin{aligned}
M_8^{i+1} = \frac{\Omega_{M8}}{2}\Big(&0.125\, M_4^i F_5^i (1-\Phi_F) + 0.25\, M_4^i F_6^i + 0.25\, M_4^i F_8^i (1-\Phi_F) + 0.5\, M_4^i F_9^i \\
&+ 0.125\, M_5^i (1-\Phi_M) F_4^i + 0.125\, M_5^i (1-\Phi_M) F_5^i (1-\Phi_F) + 0.125\, M_5^i (1-\Phi_M) F_6^i \\
&+ 0.25\, M_5^i (1-\Phi_M) F_7^i + 0.25\, M_5^i (1-\Phi_M) F_8^i (1-\Phi_F) + 0.25\, M_5^i (1-\Phi_M) F_9^i \\
&+ 0.25\, M_6^i F_4^i + 0.125\, M_6^i F_5^i (1-\Phi_F) + 0.5\, M_6^i F_7^i + 0.25\, M_6^i F_8^i (1-\Phi_F) \\
&+ 0.25\, M_7^i F_5^i (1-\Phi_F) + 0.5\, M_7^i F_6^i + 0.5\, M_7^i F_8^i (1-\Phi_F) + M_7^i F_9^i \\
&+ 0.25\, M_8^i (1-\Phi_M) F_4^i + 0.25\, M_8^i (1-\Phi_M) F_5^i (1-\Phi_F) + 0.25\, M_8^i (1-\Phi_M) F_6^i \\
&+ 0.5\, M_8^i (1-\Phi_M) F_7^i + 0.5\, M_8^i (1-\Phi_M) F_8^i (1-\Phi_F) + 0.5\, M_8^i (1-\Phi_M) F_9^i \\
&+ 0.5\, M_9^i F_4^i + 0.25\, M_9^i F_5^i (1-\Phi_F) + M_9^i F_7^i + 0.5\, M_9^i F_8^i (1-\Phi_F) + 0.25\, M_4^i F_5^i \Phi_F \\
&+ 0.5\, M_4^i F_8^i \Phi_F + 0.125\, M_5^i F_5^i \Phi_F (1-\Phi_M) + 0.125\, M_5^i F_8^i \Phi_F (1-\Phi_M) \\
&+ 0.5\, M_7^i F_5^i \Phi_F + M_7^i F_8^i \Phi_F + 0.25\, M_8^i F_5^i \Phi_F (1-\Phi_M) + 0.5\, M_8^i F_8^i \Phi_F (1-\Phi_M) \\
&+ 0.25\, M_5^i F_4^i \Phi_M + 0.125\, M_5^i F_5^i \Phi_M (1-\Phi_F) + 0.5\, M_5^i F_7^i \Phi_M \\
&+ 0.25\, M_5^i F_8^i \Phi_M (1-\Phi_F) + 0.5\, M_8^i F_4^i \Phi_M + 0.25\, M_8^i F_5^i \Phi_M (1-\Phi_F) + M_8^i F_7^i \Phi_M \\
&+ 0.5\, M_8^i F_8^i \Phi_M (1-\Phi_F)\Big) \,/\, \hat{\Omega}\,,
\end{aligned}
$$

$$
\begin{aligned}
M_9^{i+1} = \frac{\Omega_{M9}}{2}\big(&0.0625\, M_5^i (1-\Phi_M) F_5^i (1-\Phi_F) + 0.125\, M_5^i (1-\Phi_M) F_6^i \\
&+ 0.125\, M_5^i (1-\Phi_M) F_8^i (1-\Phi_F) + 0.25\, M_5^i (1-\Phi_M) F_9^i + 0.125\, M_6^i F_5^i (1-\Phi_F) \\
&+ 0.25\, M_6^i F_6^i + 0.25\, M_6^i F_8^i (1-\Phi_F) + 0.5\, M_6^i F_9^i + 0.125\, M_8^i (1-\Phi_M) F_5^i (1-\Phi_F) \\
&+ 0.25\, M_8^i (1-\Phi_M) F_6^i + 0.25\, M_8^i (1-\Phi_M) F_8^i (1-\Phi_F) + 0.5\, M_8^i (1-\Phi_M) F_9^i \\
&+ 0.25\, M_9^i F_5^i (1-\Phi_F) + 0.5\, M_9^i F_6^i + 0.5\, M_9^i F_8^i (1-\Phi_F) + M_9^i F_9^i \\
&+ 0.125\, M_5^i F_5^i \Phi_F (1-\Phi_M) + 0.125\, M_5^i F_8^i \Phi_F (1-\Phi_M) + 0.25\, M_6^i F_5^i \Phi_F \\
&+ 0.5\, M_6^i F_8^i \Phi_F + 0.25\, M_8^i F_5^i \Phi_F (1-\Phi_M) + 0.5\, M_8^i F_8^i \Phi_F (1-\Phi_M) + 0.5\, M_9^i F_5^i \Phi_F \\
&+ M_9^i F_8^i \Phi_F + 0.125\, M_5^i F_5^i \Phi_M (1-\Phi_F) + 0.25\, M_5^i F_6^i \Phi_M + 0.25\, M_5^i F_8^i \Phi_M (1-\Phi_F) \\
&+ 0.5\, M_5^i F_9^i \Phi_M + 0.25\, M_8^i F_5^i \Phi_M (1-\Phi_F) + 0.5\, M_8^i F_6^i \Phi_M + 0.5\, M_8^i F_8^i \Phi_M (1-\Phi_F) \\
&+ M_8^i F_9^i \Phi_M + 0.25\, M_5^i F_5^i \Phi_M \Phi_F + 0.5\, M_5^i F_8^i \Phi_M \Phi_F + 0.5\, M_8^i F_5^i \Phi_M \Phi_F + M_8^i F_8^i \Phi_M \Phi_F\big) \\
&/\, \hat{\Omega}\,,
\end{aligned}
$$

and an equivalent set of equations for females of each genotype. These are identical to the above except Ω_{Mj} parameters are swapped for Ω_{Fj} where $j = 1, \ldots, 9$. In these equations M_j and F_j (for $j = 1, \ldots, 9$) represent frequencies of males and females of the nine possible genotypes, Φ_M and Φ_F are the homing rates in males and females, Ω_k (for $k = M_1, \ldots, M_9, F_1, \ldots, F_9$) denotes the relative fitness of genotype k and $\hat{\Omega}$ is a normalisation factor calculated as the sum of numerators in the eighteen recursion equations (the nine shown above and their female equivalents). Parameter sets for this model under each different model configuration are shown in Supplementary Table S7.

Calculations for fitness parameters were based on Mendelian inheritance rates and the numbers of WT insects obtained from sibling crosses. We expect 25% homozygotes, 50% heterozygotes and 25% WT from *sds3*-Cas9/+ females crossed to *sds3*-Cas9/+ males. In this cross we obtained 87 WT male adults, so we would expect 87 homozygous males and 174 (87*2) heterozygous males if there was no fitness cost to the transgene. We assume there is no fitness cost to the heterozygotes and we obtained 213 fluorescent male adults. If we subtract the expected 174 heterozygotes from the 213 total we are left with 39 homozygotes. We calculated that we expect 87 homozygous and 39 is only 44.83% of that, therefore we calculate a 55% fitness cost for homozygous males. The same calculation was performed for females and the parameter values are listed in Supplementary Table S7.

Supplementary Table S7. Parameter values for each configuration of the split CRISPR gene drive. Calculations used in deriving these values are outlined in Supplementary Tables S2 and S3.

		*sds3*G1-Cas9 / *kmo*^{sgRNAs}		
Symbol	Definition	Female Value	Male Value	Source
Ω_{A+}	Relative fitness (A heterozygotes)	1.00	1.00	[1]
Ω_{AA}	Relative fitness (A homozygotes)	0.81	0.81	[1]
Ω_{B+}	Relative fitness (B heterozygotes)	1.00	1.00	This study
Ω_{BB}	Relative fitness (B homozygotes)	0.91	0.45	This study
Φ	Homing rate	0.87	0.71	This study
		bgcn-Cas9 / *kmo*^{sgRNAs}		
Ω_{A+}	Relative fitness (A heterozygotes)	1.00	1.00	[1]
Ω_{AA}	Relative fitness (A homozygotes)	0.81	0.81	[1]
Ω_{B+}	Relative fitness (B heterozygotes)	1.00	1.00	[1]
Ω_{BB}	Relative fitness (B homozygotes)	0.79	0.79	[1]
Φ	Homing rate	0.54	0.36	[1]
		nup50-Cas9 / w$^{U6b\text{-}GDe}$		
Ω_{A+}	Relative fitness (A heterozygotes)	1.000	1.000	[9]
Ω_{AA}	Relative fitness (A homozygotes)	0.900	0.900	[9]
Ω_{B+}	Relative fitness (B heterozygotes)	0.922*	1.00	[9]
Ω_{BB}	Relative fitness (B homozygotes)	0.844*	1.00	[9]
Φ	Homing rate	0.629	0.369	[9]
		*sds3*G1-Cas9 / *kmo*^{sgRNAs} (with improved fitness)		
Ω_{A+}	Relative fitness (A heterozygotes)	1.00	1.00	[1]
Ω_{AA}	Relative fitness (A homozygotes)	0.90	0.90	Assumed
Ω_{B+}	Relative fitness (B heterozygotes)	1.00	1.00	This study
Ω_{BB}	Relative fitness (B homozygotes)	0.91	0.45	This study
Φ	Homing rate	0.87	0.71	This study

**nup50*-Cas9 fitness costs take the form of fertility/fecundity reductions and are represented by a scaling f_j applied to each female genotype frequency (F_j^i).

References

1. Anderson, M. A. E. *et al.* A multiplexed, confinable CRISPR/Cas9 gene drive propagates in caged Aedes aegypti populations. *bioRxiv* (2022).
2. Edgington, M. P. & Alphey, L. S. Conditions for success of engineered underdominance gene drive systems. *J. Theor. Biol.* **430**, 128–140 (2017).
3. DAVIS, S., BAX, N. & GREWE, P. Engineered Underdominance Allows Efficient and Economical Introgression of Traits into Pest Populations. *J. Theor. Biol.* **212**, 83–98 (2001).
4. Magori, K. & Gould, F. Genetically Engineered Underdominance for Manipulation of Pest Populations: A Deterministic Model. *Genetics* **172**, 2613–2620 (2006).
5. Marshall, J. M. & Hay, B. A. Confinement of gene drive systems to local populations: A comparative analysis. *J. Theor. Biol.* **294**, 153–171 (2012).
6. Dhole, S., Vella, M. R., Lloyd, A. L. & Gould, F. Invasion and migration of spatially self-limiting gene drives: A comparative analysis. *Evol. Appl.* **11**, 794–808 (2018).
7. Unckless, R. L., Clark, A. G. & Messer, P. W. Evolution of Resistance Against CRISPR/Cas9 Gene Drive. *Genetics* **205**, 827–841 (2017).
8. Unckless, R. L., Messer, P. W., Connallon, T. & Clark, A. G. Modeling the Manipulation of Natural Populations by the Mutagenic Chain Reaction. *Genetics* **201**, 425–431 (2015).
