# Supplementary material for: Closing the gap to effective gene drive in Aedes aegypti by exploiting germline regulatory elements
Source: Nat Commun. 2023 Jan 20;14:338. doi: 10.1038/s41467-023-36029-7 (PMC9860013; doi:10.1038/s41467-023-36029-7)
Supplement: Supplementary file 2 — Reporting Summary [file 41467_2023_36029_MOESM2_ESM.pdf]

nature portfolio

Corresponding author(s): Luke Alphey

Last updated by author(s): Jan 5, 2023

Reporting Summary

Nature Portfolio wishes to improve the reproducibility of the work that we publish. This form provides structure for consistency and transparency in reporting. For further information on Nature Portfolio policies, see our Editorial Policies and the Editorial Policy Checklist.

Statistics

For all statistical analyses, confirm that the following items are present in the figure legend, table legend, main text, or Methods section.

n/a	Confirmed	
☐	☒	The exact sample size (*n*) for each experimental group/condition, given as a discrete number and unit of measurement
☐	☒	A statement on whether measurements were taken from distinct samples or whether the same sample was measured repeatedly
☐	☒	The statistical test(s) used AND whether they are one- or two-sided<br>*Only common tests should be described solely by name; describe more complex techniques in the Methods section.*
☒	☐	A description of all covariates tested
☒	☐	A description of any assumptions or corrections, such as tests of normality and adjustment for multiple comparisons
☐	☒	A full description of the statistical parameters including central tendency (e.g. means) or other basic estimates (e.g. regression coefficient) AND variation (e.g. standard deviation) or associated estimates of uncertainty (e.g. confidence intervals)
☐	☒	For null hypothesis testing, the test statistic (e.g. *F*, *t*, *r*) with confidence intervals, effect sizes, degrees of freedom and *P* value noted<br>*Give P values as exact values whenever suitable.*
☒	☐	For Bayesian analysis, information on the choice of priors and Markov chain Monte Carlo settings
☒	☐	For hierarchical and complex designs, identification of the appropriate level for tests and full reporting of outcomes
☒	☐	Estimates of effect sizes (e.g. Cohen's *d*, Pearson's *r*), indicating how they were calculated

Our web collection on statistics for biologists contains articles on many of the points above.

Software and code

Policy information about availability of computer code

Data collection	no software used
Data analysis	R version 4.0.2

For manuscripts utilizing custom algorithms or software that are central to the research but not yet described in published literature, software must be made available to editors and reviewers. We strongly encourage code deposition in a community repository (e.g. GitHub). See the Nature Portfolio guidelines for submitting code & software for further information.

Data

Policy information about availability of data

All manuscripts must include a data availability statement. This statement should provide the following information, where applicable:

- Accession codes, unique identifiers, or web links for publicly available datasets
- A description of any restrictions on data availability
- For clinical datasets or third party data, please ensure that the statement adheres to our policy

The data generated or analysed in this study are provided in the Supplementary Information/Source data file. Source data are provided with this paper.

Human research participants

Policy information about studies involving human research participants and Sex and Gender in Research.

Reporting on sex and gender	human subjects were not used in this study
Population characteristics	human subjects were not used in this study
Recruitment	human subjects were not used in this study
Ethics oversight	human subjects were not used in this study

Note that full information on the approval of the study protocol must also be provided in the manuscript.

Field-specific reporting

Please select the one below that is the best fit for your research. If you are not sure, read the appropriate sections before making your selection.

[x] Life sciences [ ] Behavioural & social sciences [ ] Ecological, evolutionary & environmental sciences

For a reference copy of the document with all sections, see nature.com/documents/nr-reporting-summary-flat.pdf

Life sciences study design

All studies must disclose on these points even when the disclosure is negative.

Sample size	A basic power analysis was performed and determined that for 0.8 power, we should analyse at least 200 for a 10% difference between inheritance rates.
Data exclusions	No data were excluded
Replication	Biological replicates were performed as described in the main article text. Experimental crosses were performed with 10 or more F1 individuals of the specified genotype/phenotype simultaneously, each distinct pairing acting as a biological replicate. The progeny were collected as a pool and screened, averaging the data across all replicates, or collected from each female individually and screened. No replicates were excluded.
Randomization	Larvae/pupae were screened and separated based on genotype (indicated by the presence of fluorescent markers) then individuals were randomly selected for experiments from this pool.
Blinding	Blinding was not performed as none of the data collected were subjective.

Reporting for specific materials, systems and methods

We require information from authors about some types of materials, experimental systems and methods used in many studies. Here, indicate whether each material, system or method listed is relevant to your study. If you are not sure if a list item applies to your research, read the appropriate section before selecting a response.

Materials & experimental systems

n/a	Involved in the study	
[x]	[ ]	Antibodies
[x]	[ ]	Eukaryotic cell lines
[x]	[ ]	Palaeontology and archaeology
[ ]	[x]	Animals and other organisms
[x]	[ ]	Clinical data
[x]	[ ]	Dual use research of concern

Methods

n/a	Involved in the study	
[x]	[ ]	ChIP-seq
[x]	[ ]	Flow cytometry
[x]	[ ]	MRI-based neuroimaging

Animals and other research organisms

Policy information about studies involving animals; ARRIVE guidelines recommended for reporting animal research, and Sex and Gender in Research

Laboratory animals	Aedes aegypti Liverpool strain, males and females at all ages, crosses were performed with 3-7 day post-eclosion adults, screening was performed at larvae/pupae stages.
Wild animals	No wild animals were used in this study
Reporting on sex	Data was collected for males and females separately and is reported as such. Some differences were observed in the inheritance bias depending on the sex of the parent carrying Cas9 and this was closely tracked and is reported in the manuscript.
Field-collected samples	No field collected samples were used.
Ethics oversight	No ethical oversight is required for insect species

Note that full information on the approval of the study protocol must also be provided in the manuscript.
